# Genome-wide profiling of DNA methylation and gene expression identifies candidate genes for human diabetic neuropathy

**DOI:** 10.1186/s13148-020-00913-6

**Published:** 2020-08-12

**Authors:** Kai Guo, Stephanie A. Eid, Sarah E. Elzinga, Crystal Pacut, Eva L. Feldman, Junguk Hur

**Affiliations:** 1 Department of Biomedical Sciences, School of Medicine and Health Sciences, University of North Dakota, 1301 North Columbia Rd. Stop 9037, Grand Forks, ND 58202-9037 USA; 2 Department of Neurology, School of Medicine, University of Michigan, Ann Arbor, MI 48109 USA

**Keywords:** Type 2 diabetes, Diabetic peripheral neuropathy, Transcriptomics, Epigenetics, DNA methylation, Gene expression, HbA1c, Human

## Abstract

**Background:**

Diabetic peripheral neuropathy (DPN) is the most common complication of type 2 diabetes (T2D). Although the cellular and molecular mechanisms of DPN are poorly understood, we and others have shown that altered gene expression and DNA methylation are implicated in disease pathogenesis. However, how DNA methylation might functionally impact gene expression and contribute to nerve damage remains unclear. Here, we analyzed genome-wide transcriptomic and methylomic profiles of sural nerves from T2D patients with DPN.

**Results:**

Unbiased clustering of transcriptomics data separated samples into groups, which correlated with HbA1c levels. Accordingly, we found 998 differentially expressed genes (DEGs) and 929 differentially methylated genes (DMGs) between the groups with the highest and lowest HbA1c levels. Functional enrichment analysis revealed that DEGs and DMGs were enriched for pathways known to play a role in DPN, including those related to the immune system, extracellular matrix (ECM), and axon guidance. To understand the interaction between the transcriptome and methylome in DPN, we performed an integrated analysis of the overlapping genes between DEGs and DMGs. Integrated functional and network analysis identified genes and pathways modulating functions such as immune response, ECM regulation, and PI3K-Akt signaling.

**Conclusion:**

These results suggest for the first time that DNA methylation is a mechanism regulating gene expression in DPN. Overall, DPN patients with high HbA1c have distinct alterations in sural nerve DNA methylome and transcriptome, suggesting that optimal glycemic control in DPN patients is an important factor in maintaining epigenetic homeostasis and nerve function.

## Background

Type 2 diabetes (T2D) accounts for 90 to 95% of the 463 million people suffering from diabetes worldwide [1] and is associated with macro- and microvascular complications. Of these, microvascular complications are more common and affect multiple tissues including the eye, kidney, and nerve [2, 3]. Diabetic neuropathy affects the nerve and is the most prevalent microvascular complication that can present in multiple forms, the most common being diabetic peripheral neuropathy (DPN) [4]. DPN affects up to 60% of T2D patients and is characterized by distal-to-proximal nerve damage that results in reduced sensation, chronic pain, increased infection risk, and recurrent foot ulceration that can lead to lower limb amputations [4–6]. Despite the enormity of the problem, current treatments often fail to slow or reverse DPN progression in T2D [7]. Therefore, understanding the pathogenesis of DPN is critical for developing mechanism-based therapies.

The risk of developing T2D and DPN is determined by both genetic and lifestyle factors [3, 8]. Transcriptome profiling has provided insight into disease pathogenesis by identifying numerous genes and pathways implicated in DPN, including inflammation [9–12], oxidative stress [10, 12, 13], lipid metabolism [14, 15], and mitochondrial dysfunction [11, 16]. However, many of these previous analyses were either performed in animal models or using microarrays, which have several limitations, including a narrow dynamic range, low sensitivity, and dependency on predefined transcripts [17–19]. Next-generation RNA-sequencing (RNA-seq) is considered a potential alternative to microarrays because it is unbiased and more sensitive [18, 20]. However, RNA-seq has not yet been used to characterize the transcriptomic changes in human DPN.

In addition to genetic factors, epigenetic mechanisms, such as DNA methylation, histone modifications, chromatin remodeling, and RNA editing and biogenesis have recently emerged as a potential link between gene expression and environmental factors [21]. DNA methylation refers to the reversible attachment of a methyl group to a cytosine within cytosine–phosphate–guanine (CpG) dinucleotides [22]. In differentiated cells, DNA methylation contributes to the maintenance of normal DNA structure, chromosome stability, and gene regulation [23]. DNA methylation regulates gene expression without altering the underlying DNA sequence and is of particular interest because of its emerging role in T2D and its complications [24–27]. We recently showed that aberrant DNA methylation is involved in nerve degeneration in T2D and DPN in a small cohort of patients [24]. Specifically, our results highlighted the role of DNA methylation in regulating pathways previously shown to be implicated in DPN pathogenesis, including axon guidance, glycerophospholipid metabolism, and MAPK signaling. However, much less is known about the impact of differential DNA methylation on gene expression in DPN and how the interaction between genetic and epigenetic mechanisms may affect biological pathways during DPN pathogenesis.

In this study, we expanded on our previous findings by conducting a comprehensive systems biology analysis that combines global DNA methylome and genome-wide transcriptome profiling in human sural nerves of T2D patients with DPN. By integrating epigenomic and transcriptomic data, we found associations between hemoglobin A1c (HbA1c), DNA methylation, and differential gene expression patterns within pathways known to play a key role in DPN pathogenesis. These findings support the involvement of epigenetic dysregulation in DPN and offer candidate targets for developing therapeutic strategies for DPN.

## Results

### Study population

To identify specific mechanisms that are differentially transcribed in T2D and DPN, we determined gene expression profiles using RNA-seq in sural nerve biopsies obtained from DPN patients from a double-blind placebo-controlled clinical trial of a candidate treatment that proved ineffective [28, 29]. Sural nerve biopsies (*n* = 78) from T2D patients with DPN were initially classified based on changes in myelinated fiber density (MFD) over 52 weeks [30]. Genome-wide DNA methylation profiling was also performed on the same cohort, using reduced representation bisulfite sequencing (RRBS). Patient clinical characteristics are available in Additional file 2: Table S1, including HbA1c, triglyceride and cholesterol levels, and neuropathy phenotyping by MFD counts and O’Brien neuropathy score.

### RNA-seq and RRBS quality assessment and alignment summary

All samples had high-quality RNA-seq reads with an average of 96% reads per sample that survived trimming with Trimmomatic (Additional file 2: Table S2). Approximately 77% of the total reads uniquely aligned to the hg19 reference human genome, except one sample (ID 63096) with a unique mapping rate of 33%, which was excluded from subsequent analyses. In total, an average of 90 million paired-end and single-end reads per sample was generated by RNA-seq. To identify genes and pathways under epigenetic control in human DPN, we next examined genome-wide DNA methylation using RRBS profiling. Briefly, over 98% were high-quality reads, and approximately 52 million reads per sample could be uniquely mapped (55%~68%) to hg19 (Additional file 2: Table S2). CpG sites on X and Y chromosomes were excluded, resulting in a total of 1,602,934 CpG sites identified for differential methylation analysis. In total, an average of 45 million pairs of 50-bp paired-end reads and 90 million 50-bp single-end reads were generated for RNA-seq and RRBS, respectively.

### Data-driven clustering analysis

**Fig. 1:**
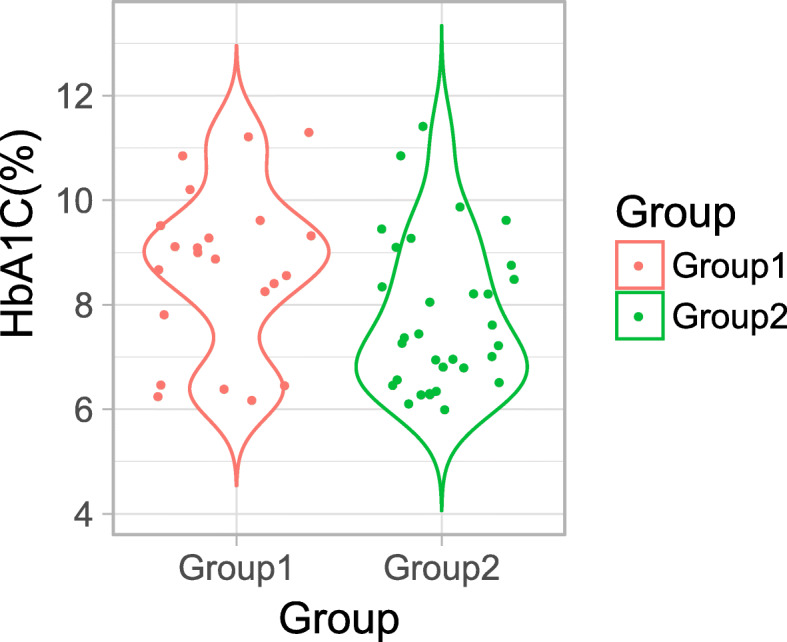
Violin plot of % hemoglobin A1c (HbA1c) level distribution in groups 1 and 2. Each dot corresponds to the HbA1c (%) of a patient and color corresponds to the groups

We first examined the overall gene expression similarity among the samples using hierarchical clustering analysis. Cluster dendrogram (Additional file 1: Figure S1A) defined three groups of samples, which did not correlate with the previous classification based on changes in MFD [30]. We then identified the clinical factors that were significantly associated with these transcriptomics data-driven groups by applying principal component analysis (Additional file 1: Figure S1B) and multifactorial logistic regression analysis. HbA1C levels were the only clinical factor that significantly differed across these groups (*p* = 0.04; Fig. 1). For downstream analyses, we focused on clusters with the highest (group 1, *n* = 21) and lowest (group 2, *n* = 32) HbA1c. Clinical characteristics and neuropathy measures for groups 1 and 2 are summarized in Table 1.

**Table 1:** Clinical characteristics and neuropathy measures of groups 1 and 2

Group	Group 1 (***n*** = 21)	Group 2 (***n*** = 32)	***p*** value
Age	57.4 ± 9.1	55.7 ± 7.6	0.46
Sex (male/female)	13/8	20 /12	0.77
Body mass index (kg/m^2^)	30.1 ± 6.0	31 ± 5.6	0.55
HbA1c (%)	8.7 ± 1.6	7.8 ± 1.4	0.04*
Cholesterol (mmol/L)	5.7 ± 1.4	5.9 ± 1.1	0.56
Triglyceride (mmol/L)	2.8 ± 1.8	2.8 ± 2.5	0.96
Diabetes duration (years)	8.6 ± 6.9	9.9 ± 6.2	0.50
MFD, baseline (fibers/mm^2^)	3783.2 ± 1714.9	3073.7 ± 1567.3	0.14
MFD, 52 weeks (fibers/mm^2^)	3771.1 ± 1965.9	2942.4 ± 1750.2	0.13
MFD, change (fibers/mm^2^)	−12.1 ± 1062.2	−131.3 ± 1082.1	0.69
ALCAR treated, *n* (%)	15 (71.4%)	20 (62.5%)	0.56

Values are the mean ± standard deviation (SD). *p* value was calculated by Student’s *t* test and Fisher’s exact test

*ALCAR* acetyl-L-carnitine, *MFD* myelinated fiber density

*p value < 0.05

### Differentially expressed genes (DEGs) analysis

A total of 998 genes were differentially expressed in group 1 (high HbA1c) relative to group 2 (low HbA1c) with a Benjamini-Hochberg adjusted *p* value < 0.01. Of the 998 DEGs, we found that 542 genes were significantly upregulated and 456 genes were significantly downregulated (Additional file 2: Table S3). The top 30 most upregulated and downregulated DEGs along with their annotation are presented in Table 2. Additionally, the top 50 most significant DEGs are labeled in an MA plot (Additional file 1: Figure S2) and gene expression levels of the 100 most significant DEGs in group 1 versus group 2 are displayed in a heatmap (Additional file 1: Figure S3). Interestingly, significantly upregulated genes in group 1 compared to group 2 included the long noncoding RNA *NEAT1*, which has been recently implicated in T2D and neurodegeneration [31, 32]. The immune response has also been repeatedly shown to be involved in T2D and neurodegeneration [9, 14], and downregulated DEGs included immune response genes *CCDC80* and *CD14*.

**Table 2:** The 15 most upregulated and downregulated DEGs

Gene symbol	Entrez ID	Annotation	Log_**2**_FoldChange	Adjusted ***p*** value
RPS29	6189	Ribosomal protein S3A	1.51	1.50E−06
ANKRD36B	57730	Ankyrin repeat domain 36B	1.30	1.64E−07
ANKRD36	375248	Ankyrin repeat domain 36	1.29	7.32E−11
SPTBN5	51332	Spectrin beta, non-erythrocytic 5	1.29	6.03E−06
SLC7A5P2	27173	Solute carrier family 39 member 1	1.21	1.84E−07
HERC2P2	400322	Hect domain and RLD 2 pseudogene 2	1.18	1.67E−07
MIR143HG	728264	Cardiac mesoderm enhancer-associated noncoding RNA	1.18	2.62E−06
KAT2A	2648	Lysine acetyltransferase 2A	1.17	5.24E−08
CCNL2	81669	Cyclin L2	1.15	4.74E−13
MYO15B	55930	Myosin VC	1.14	3.50E−09
LOC440300	440300	Chondroitin sulfate proteoglycan 4 pseudogene	1.14	5.28E−08
LINC00342	378805	Long intergenic non-protein coding RNA, p53-induced transcript	1.13	4.40E−08
LENG8	114823	Leukocyte receptor cluster member 8	1.12	1.92E−10
SMG1P3	100271836	SMG1P3, nonsense mediated mRNA decay associated PI3K related kinase pseudogene 3	1.12	2.94E−08
NEAT1	283131	Nuclear paraspeckle assembly transcript 1 (non-protein coding)	1.11	1.52E−11
ASPN	54829	Asporin	−1.11	1.68E−06
PI16	221476	Peptidase inhibitor 16	−1.12	3.13E−06
SELPLG	6404	Selectin P ligand	−1.14	3.32E−04
WISP2	8839	WNT1 inducible signaling pathway protein 2	−1.16	4.30E−07
MRC1	4360	Mannose receptor C-type 1	−1.19	2.10E− 05
SELL	6402	Selectin L	−1.19	3.22E−04
CYBB	1536	Cytochrome b-245 beta chain	−1.22	1.74E−05
CCDC80	81576	Coiled-coil domain containing 130	−1.23	1.93E−12
C1QA	712	Complement C1q A chain	−1.23	1.24E−04
LYVE1	10894	Lymphatic vessel endothelial hyaluronan receptor 1	−1.28	2.11E−06
MPEG1	219972	Macrophage expressed 1	−1.31	2.06E−07
CD14	920	CD4 molecule	−1.36	4.02E−07
F13A1	2162	Coagulation factor XIII A chain	−1.40	1.00E−08
C1QC	714	Complement C1q C chain	−1.45	1.45E−08
C1QB	713	Complement C1q B chain	−1.50	6.92E−08

### Functional enrichment analyses of DEGs

**Fig. 2:**
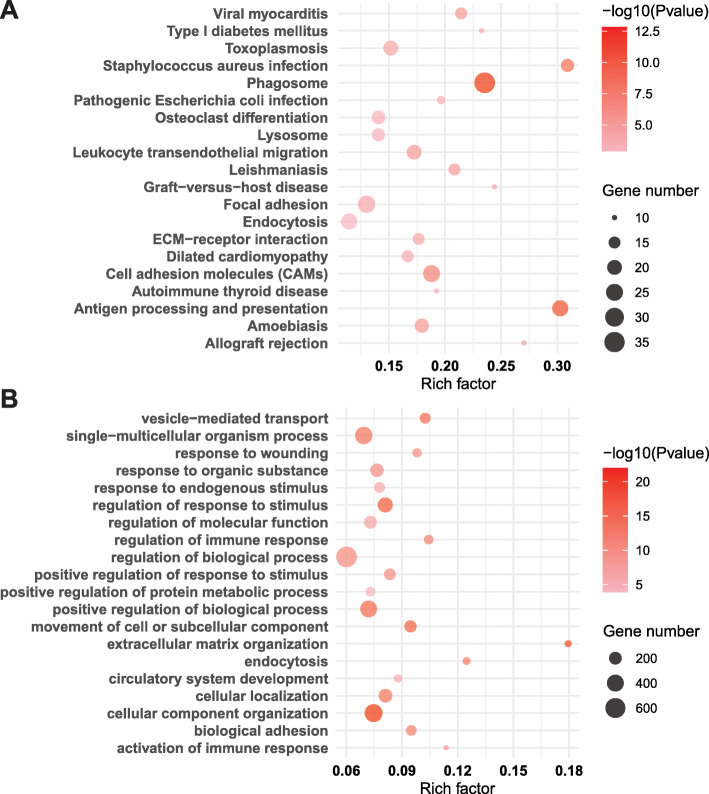
Functional enrichment analysis of DEGs by KEGG and GO. The 20 most significantly enriched biological functions using KEGG (**a**) and GO (**b**) are illustrated in dot plots. Rich factor refers to the proportion of DEGs belonging to a specific term. Node size (gene number) refers to the number of DEGs within each term and node color indicates the level of significance (−log_10_ *p* value)

To better understand biological functions and molecular pathways associated with the DEGs, we performed functional enrichment analysis using Kyoto Encyclopedia of Genes and Genomes (KEGG) pathway, Gene Ontology (GO), Reactome, and Disease Ontology (DO) terms with an adjusted *p* value < 0.05 as the significance cutoff. Using KEGG pathway analysis, we found significant enrichment of pathways related to phagosomes as well as antigen processing and presentation (Fig. 2a, Additional file 2: Table S4). “Cell adhesion molecules (CAMs),” “Type I diabetes mellitus,” and “Neurotrophin signaling pathway” were also significantly enriched (Fig. 2a, Additional file 2: Table S4). Moreover, 688 GO terms were significantly overrepresented in group 1 relative to group 2. Further clustering based on gene content using Database for Annotation, Visualization and Integrated Discovery (DAVID) clustering [33] identified 24 significant GO clusters. Those included biological processes such as “cellular component organization,” “extracellular matrix organization,” and “regulation of immune response” (Fig. 2b, Additional file 2: Table S5). Within these pathways, top DEGs included members of the transforming growth factor-beta (TGF-β) family, previously associated with the development of DPN [34]. Additionally, pathways involving calcium signaling and homeostasis were dysregulated in group 1 compared to group 2, with significant enrichment in terms like “calcium-mediated signaling” and “calcium ion transport into cytosol” (Additional file 2: Table S5).

We also performed enrichment analysis using Reactome and DO, and found that 58 Reactome and 39 DO pathways were significantly altered (adjusted *p* value < 0.05, Additional file 1: Figure S4A-B, Additional file 2: Tables S6-7). Many of these pathways were similar to those identified by KEGG and GO analysis, including “adaptive immune system,” “extracellular matrix organization,” “axon guidance,” and “demyelinating disease.” Interestingly, DEGs were highly involved in neuronal and glial function as well as myelination according to DO, such as insulin-like growth factor I (*IGF-I*) (Additional file 2: Tables S6-7) and the antioxidant superoxide dismutase 2 (*SOD2*) (Additional file 2: Table S7) [35, 36].

### DNA methylation changes

**Fig. 3:**
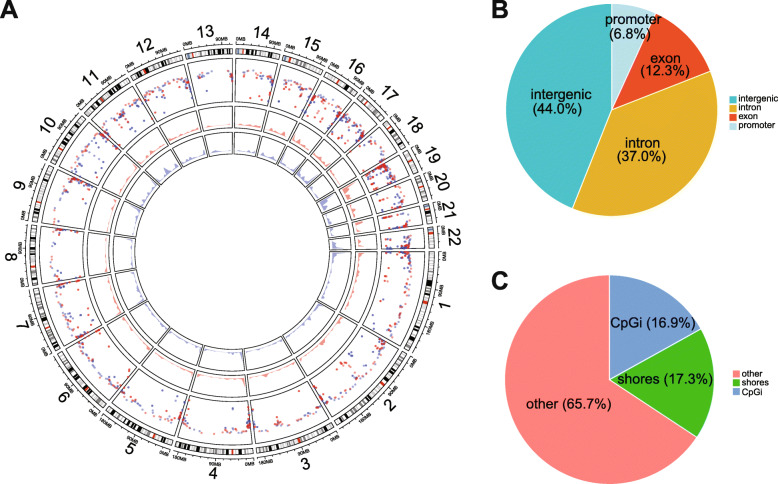
Distribution of differentially methylated CpGs (DMCs). **a** DMCs across chromosomes are depicted in a circular plot. Hyper- and hypomethylated CpGs are colored in red and blue, respectively, relative to group 2. The distributions of DMCs summarized based on genomic location (**b**) and relative to CpG islands (CpGi) (**c**)

We found a total of 2066 differentially methylated CpG sites (DMCs) between group 1 and group 2, of which 1169 were hypomethylated and 897 were hypermethylated (Additional file 2: Table S8). The overall percentage of DMCs ranged between 45 and 50% (Fig. 3a), with no significant differences in methylation patterns across the chromosomes. We also determined the genomic distribution of altered DNA methylation and the distribution of DMCs in relation to CpG islands. Approximately 7% of DMCs were located in promoter regions, 37% in introns, 12% in exons, and ~ 44% in intergenic regions (Fig. 3b). Additionally, the majority of DMCs were in “non-CpG-rich regions” (denoted as “other” in the figure), while 34% of DMCs were located in CpG islands and shores (Fig. 3c).

To explore how DMCs induce functional changes in the genome, we mapped them to 1519 unique NCBI Reference Sequence Database (RefSeq) IDs (Additional file 2: Table S8). Only the genes with valid official gene symbols, referred to as differential methylated genes (DMGs) (*n* = 1489), were included in downstream analyses. A total of 929 DMGs (mapped from 1167 DMCs) exhibited hypomethylation, while 686 DMGs (mapped from 894 DMCs) exhibited hypermethylation in group 1 compared to group 2. Approximately 8% of DMGs (*n* = 126) were mapped from both hypermethylated and hypomethylated DMCs. With respect to the distance of DMCs from transcript start sites (TSSs), 201 DMCs corresponding to 153 DMGs were within a 5 kb distance upstream from their respective TSSs. The top 20 DMCs within the promoter regions (< 5 kb distance from TSS), along with their annotated genes (DMGs), are listed in Table 3 and include several microRNAs and coding genes.

**Table 3:** Top 20 DMCs within the promoter region

Gene	CpG location	***q*** value	Methylation difference (%)	Type	Gene description
DOK7	chr4:3486002	2.17E−38	−50.10	Protein_coding	Docking protein 7
MIR3648-1	chr21:9825780	1.02E−20	−30.84	MicroRNA	MicroRNA 3648-1
GALNT9	chr12:132692165	2.53E−33	28.53	Protein_coding	Polypeptide N-acetylgalactosaminyltransferase 9
C15orf57	chr15:40858818	5.12E−24	28.46	Protein_coding	Chromosome 15 open reading frame 57
TNNT3	chr11:1939527	5.15E−08	−28.28	Protein_coding	Troponin T type 3 (skeletal, fast)
P2RX1	chr17:3821014	4.19E−34	−27.10	Protein_coding	Purinergic receptor P2X, ligand-gated ion channel, 1
LMF1	chr16:1022800	2.21E−11	−26.96	Protein_coding	Lipase maturation factor 1
VAC14-AS1	chr16:70787597	7.18E−07	−26.42	Antisense	VAC14 antisense RNA 1
HOXA-AS3	chr7:27179251	4.33E−06	−25.87	Antisense	HOXA cluster antisense RNA 3
LOC100129931	chr4:7048841	1.02E−58	25.30	ncRNA	Uncharacterized LOC100129931
LINC01168	chr10:134778467	1.01E−13	−25.27	LincRNA	Long intergenic non-protein coding RNA 1168
SNORD59B	chr12:57038157	1.58E−05	24.49	snoRNA	Small nucleolar RNA, C/D box 59B
SNAI3	chr16:88753392	5.22E−06	−24.27	Protein_coding	Snail homolog 3 (Drosophila)
MIR3648-1	chr21:9825613	1.59E−33	24.04	MicroRNA	MicroRNA 3648-1
MIR3687-1	chr21:9826027	2.08E−17	−24.02	MicroRNA	MicroRNA 3687-1
LOC100134317	chr19:36800758	5.25E−20	23.97	ncRNA	Uncharacterized LOC100134317
MIR663AHG	chr20:26189971	1.66E−07	23.77	MicroRNA	MIR663A host gene
AEBP2	chr12:19592255	2.69E−07	23.69	Protein_coding	AE binding protein 2
CABLES1	chr18:20712894	1.01E−05	23.43	Protein_coding	Cdk5 and Abl enzyme substrate 1
GBP5	chr1:89739007	1.41E−35	22.80	Protein_coding	Guanylate binding protein 5

### Functional enrichment analysis of DMGs

**Fig. 4:**
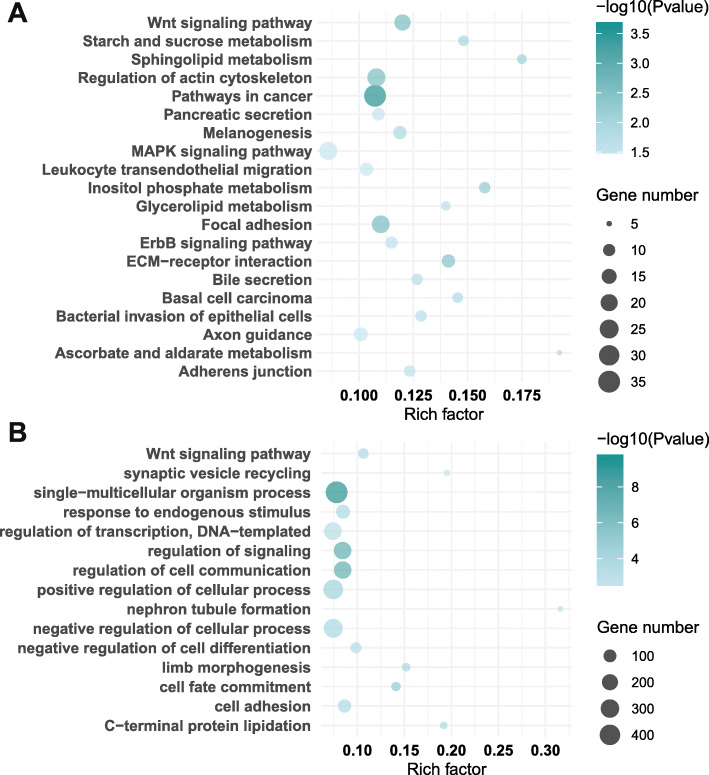
Functional enrichment analysis of DMGs by KEGG and GO. The 20 most significantly enriched biological functions using KEGG (**a**) and GO (**b**) are illustrated in dot plots. Rich factor refers to the proportion of DMGs belonging to a specific term. Node size (gene number) refers to the number of DEGs within each term and node color indicates the level of significance (−log_10_ *p* value)

We next annotated DMGs for biological and functional enrichment using KEGG, GO, Reactome, and DO terms (Fig. 4, Additional file 1: Figure S5). A total of 24 KEGG pathways were significantly enriched and included “extracellular matrix (ECM)-receptor interaction,” “MAPK signaling pathway,” “ErbB signaling pathway,” and “axon guidance” (Fig. 4a, Additional file 2: Table S9). Significant DMGs within these pathways included structural genes and genes related to nerve damage, inflammation, and diabetes, such as *ERBB4* and *IL1B*. GO analysis showed that 1519 DMGs were highly enriched in 929 biological processes, which were further clustered using DAVID clustering into 15 GO clusters with kappa > 0.5. Over-represented clusters included processes related to ECM remodeling such as cell adhesion as well as nervous system development (Fig. 4b, Additional file 2: Table S10), with significant DMGs such as *SOX9* and *Nr2f2* (Additional file 2: Table S10).

Additionally, we performed a pathway enrichment analysis using the Reactome database and DO analysis, and identified 117 and 55 significant pathways, respectively (Additional file 1: Figure S5). Enriched Reactome pathways primarily included those involved in insulin signaling such as “PI5P, PP2A and IER3 regulate PI3K/Akt signaling,” “PI3K cascade,” “IRS-mediated signaling,” and “IRS-related events triggered by IGF1R” (Additional file 1: Figure S5A, Additional file 2: Table S11). DMGs under Reactome were also associated with “VEGFA-VEGFR2 pathway” (Additional file 2: Table S11).

### Overlap analysis between DMGs and DEGs

**Fig. 5:**
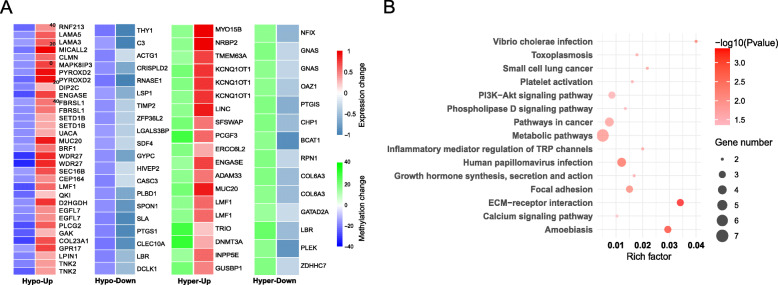
Overlap between DMGs and DEGs. **a** Heatmap of methylation and expression changes of the overlapping DMGs and DEGs. The first column of each group corresponds to the methylation change (green: hypermethylated, blue: hypomethylated), while the second column represents the gene expression change (red: upregulated, blue: downregulated). DMCs belonging to the same genes are illustrated separately. **b** Dots include the significantly enriched KEGG pathways among the overlapping genes with opposite directions between DMGs and DEGs (hypo-up and hyper-down groups)

To understand the relationship between epigenetic regulation and transcriptomic changes in DPN and T2D, we examined the overlap between DMGs and DEGs. To that end, we screened 998 DEGs (Additional file 2: Table S3) and 1489 DMGs (Additional file 2: Table S8) for both altered gene expression and differential methylation, along with their corresponding directional changes. We found that 71 genes were shared between the DMGs and DEGs sets (Additional file 2: Table S13), and the majority of corresponding DMCs were located in intronic or intergenic regions. Among these 71 shared genes, 13 genes included multiple DMCs, while 58 genes included a single DMC per gene. These shared genes were divided into four categories based on the directional changes in DNA methylation and gene expression: “Hypo-Down” for the hypomethylated and downregulated genes (*n* = 20), “Hypo-Up” for the hypomethylated and upregulated genes (*n* = 33), “Hyper-Down” for the hypermethylated and downregulated genes (*n* = 14), and “Hyper-Up” for the hypermethylated and upregulated genes (*n* = 19) (Fig. 5a).

**Fig. 6:**
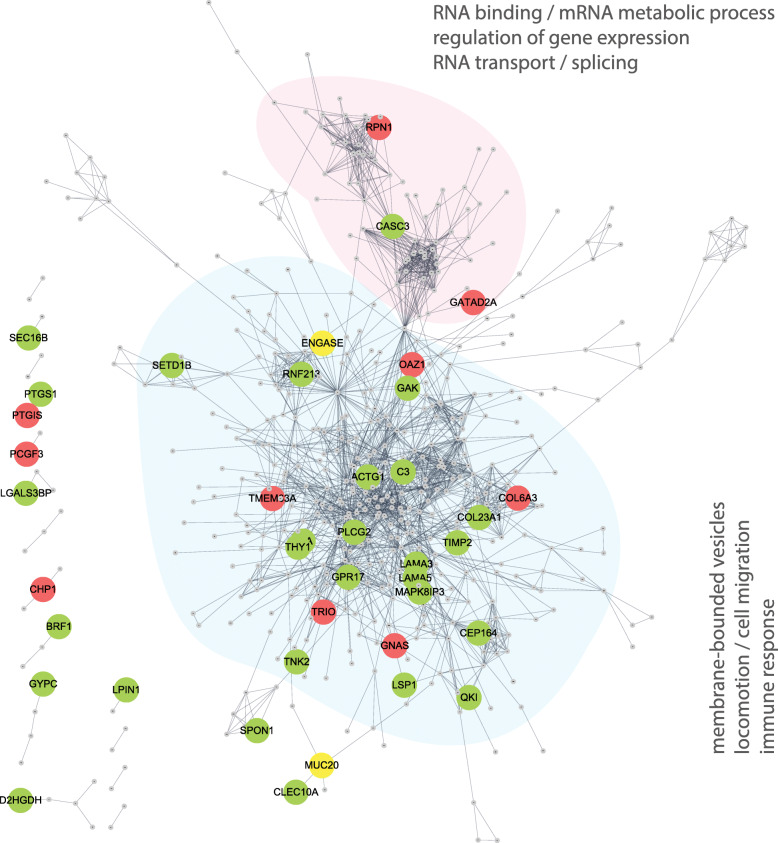
Interaction network of DMGs and DEGs. An interaction network among the DEGs was generated using STRING, a database of known and predicted protein-protein interactions with the highest confidence score cutoff (score > 0.9). Node corresponds to a DEG, while edge indicates an interaction between two nodes. To visualize DMGs superimposed on the gene interaction network, only the DEGs with corresponding significant change in DNA methylation are labeled and highlighted with the color indicating the direction of methylation change: hypermethylation (red), hypomethylation (green), and both hyper- and hypomethylation (yellow). Major clusters were subjected to functional enrichment analysis in terms of GO and KEGG/Reactome pathways, with the most significantly enriched biological functions annotated in the network. Singletons not connected to another gene were excluded from this network

To further explore the interaction between DEGs and DMGs, we first constructed a gene interaction network using DEGs to identify gene sets that might highly interact with each other. DEGs were queried against the Search Tool for the Retrieval of Interacting Genes (STRING) database, and we generated an interaction network using the highest scoring confidence cutoff (score > 0.9). DMGs were then superimposed on the gene interaction network, and only the DEGs with a corresponding significant change in DNA methylation were listed (Fig. 6). This network included two large subnetworks and multiple small ones. The largest subnetwork included genes highly related to the immune response and locomotion and cell migration, and the second-largest subnetwork included genes involved in RNA binding and regulation of gene expression. Network analysis further showed a high degree of connectivity between phospholipase C gamma 2 (*PLCG2*) and G protein-coupled receptor 17 (*GPR17*), suggesting a role in DPN pathogenesis. Importantly, the network also highlighted highly connected overlapping genes between the differentially expressed and methylated gene sets, such as Thy-1 cell surface antigen (*Thy1*), also known as CD90*. Thy1* has been implicated in nervous system development, neuronal injury, and immune response [37], and was found to be hypomethylated and downregulated in group 1 relative to group 2. Our results also show that mitogen-activated protein kinase 8 interacting protein 3 (*MAPK8IP3*), recently implicated in nerve degeneration and axonal neuropathy [38], is hypomethylated and upregulated in group 1 compared to group 2.

We lastly performed functional enrichment analysis on the genes showing opposite direction (Fig. 5b) and same direction (Additional file 1: Figure S6) of change in DNA methylation and gene expression. KEGG analysis showed that these genes are highly enriched in pathways including “ECM-receptor interaction,” “Pathways in cancer,” “PI3K−Akt signaling pathway,” and “calcium signaling pathway.” These numerous epigenetically regulated pathways are in agreement with our previous study, which demonstrated that many of the pathways previously identified as key mechanisms in human DPN are under epigenetic control [24].

### Experimental validation using RT-qPCR

**Fig. 7:**
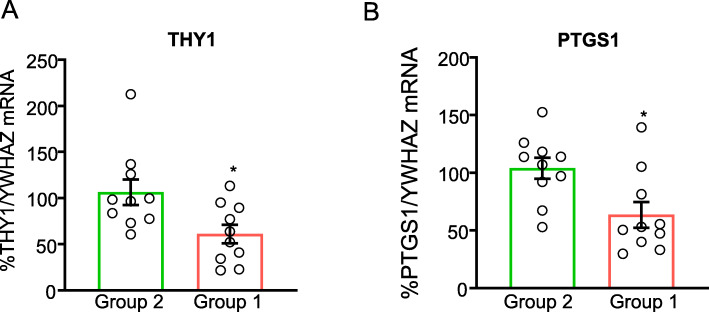
qPCR validation of genes with a similar direction of change in DNA methylation and gene expression. RNA was extracted from human sural nerve biopsies from group 1 (*n*  =  9–10) and group 2 (*n*  =  10), which was used as the relative control group (set to 100%), and quantified by RT-qPCR. Data were normalized to tyrosine 3-monooxygenase/tryptophan 5-monooxygenase activation protein zeta (YWHAZ) and expressed as % change calculated by the 2^−ΔΔCT^ method. *p < 0.05 relative to group 2

Based on biological relevance, we next selected two targets from the DMG and DEG overlap and functional enrichment analysis to validate by real-time quantitative PCR (RT-qPCR) (Fig. 7): *Thy1* and prostaglandin-endoperoxide synthase 1 (*PTGS1*), with CpG sites located within intergenic regions. *Thy1* was significantly hypomethylated by RRBS and downregulated by RNA-seq, and its gene expression followed a similar decreasing pattern in group 1 compared to group 2 (*p* = 0.0163; Fig. 7a). The directions of methylation and expression changes for *PTGS1*, a gene involved in neuropathic pain [39], were also discordant (hypomethylated/downregulated), and this finding was further confirmed by RT-qPCR, with a significant decrease in *PTGS1* gene expression in group 1 versus group 2 (*p* = 0.0111; Fig. 7b).

## Discussion

Although several genes and pathways are implicated in the pathophysiology of DPN, the molecular mechanisms underlying disease onset and progression remain largely unknown. DNA methylation, a major regulator of gene expression, has recently emerged as a key player in the development of diabetic complications, including DPN [24–27]. The aim of the present study was therefore to define the methylomic and transcriptomic signatures of human DPN and to understand how DNA methylation influences gene expression and thus contributes to nerve fiber damage. Using RNA-seq and RRBS, we determined differentially altered molecular pathways in both the methylome and transcriptome of sural nerves from well-characterized patients with T2D and DPN, and confirm findings gathered from experimental and clinical DPN [10, 24, 40, 41]. We also report the first integrated analysis of methylomic and transcriptomic datasets and identified differences in the methylation of genes encoding pathways involved in “immune response,” “ECM-receptor interaction,” and “PI3K−Akt signaling pathway.” These findings not only shed light on the role of epigenetic mechanisms in driving the expression of well-known regulators and novel targets of DPN but also help identify potential disease-modifying targets.

DPN is characterized by decreased nerve conduction velocities and the loss of both unmyelinated and myelinated nerve fibers [30, 40]. Accordingly, our initial analysis of the present cohort classified sural nerve biopsies based on their MFD, to reflect disease progression [30]. In this study, unbiased clustering of RNA-seq data separated those samples into distinctive clusters, which significantly differed in HbA1c levels. T2D patients with high HbA1c (8.7 ± 1.6%) showed distinct variations in their sural nerve transcriptome relative to patients with lower HbA1c (7.8 ± 1.4%). These findings suggest that transcriptomic changes in this cohort are associated with glycemic status, an established and independent risk factor for DPN development [30, 42].

When looking at transcriptomic changes, we found alterations in the expression of genes involved in immune response, calcium signaling, and axon guidance, which are highly relevant to nerve injury based on our current understanding of DPN pathogenesis [40, 43]. Moreover, the downregulation of the antioxidant *SOD2* was of particular interest because this effect has been previously associated with increased oxidative damage and a stronger neuropathy phenotype in animal DPN models [44]. Consistent with these findings, the downregulated *SOD2* suggests that antioxidant capacity is depleted in sural nerve biopsies of T2D patients with higher HbA1c and may participate in hyperglycemia-induced nerve injury. HbA1c-related nerve injury was also accompanied by changes in DNA methylation that mostly occurred within the gene body, a common pattern of diabetic complications [45]. DMGs were highly enriched for functions related to MAPK signaling, axon guidance, and VEGFA-VEGFR2 pathway. These observations, in line with our previous findings in a smaller human cohort and animal models of DPN, suggest a direct role for these pathways in DPN progression [10, 24].

Although RNA-seq and RRBS revealed transcriptomic and methylomic changes in sural nerve biopsies, this approach alone did not identify specific genes whose expression levels may be influenced by epigenetic factors. For the first time, we integrated methylation and gene expression datasets to determine whether the interaction between the methylome and transcriptome differed in T2D DPN patients with high (poor glycemic control) versus low HbA1c (good glycemic control). Specifically, our results determined that DNA methylation within the promoter or gene body was both concordantly and discordantly associated with gene expression. DNA methylation within promoter regions is associated with gene silencing and is generally considered a hallmark of cancer [21]. However, results from our group and others show that DNA methylation changes, in particular discordant changes, occur more frequently within gene bodies in diabetic complications, including DPN [24, 45]. Here, we confirmed and expanded our previous findings and demonstrated that the direction of change between DNA methylation and gene expression in human nerves can be both concordant and discordant.

Network analysis of overlapping DMG and DEG genes identified pathways involved in immune response, an important player in DPN development [46]. However, our results extend the literature to identify for the first time the role of DNA methylation in regulating immune-associated gene expression in human DPN. We found that *PLCG2* was hypomethylated and overexpressed in sural nerves from patients with higher HbA1c. *PLCG2* encodes a signaling protein essential for regulating immune cells, including macrophages [47], and has been recently implicated in neurodegenerative disorders [48, 49]. Peripheral nerve tissue consists of multiple cell types, including immune system macrophages and supportive Schwann cells, the myelinating cells of the peripheral nervous system, and axonal extensions. The current analysis did not account for the differential cell-type composition of sural nerve biopsies between groups 1 and 2. Therefore, it is unclear whether *PLCG2* was expressed by the Schwann cells or infiltrating macrophages from the nerve biopsy tissue. However, evidence suggests that these two cell types interact with each other in the context of nerve injury and demyelination, and a similar mechanism may be occurring in human DPN in a *PLCG2*-associated manner [50, 51]. Future experiments addressing cell-specific changes would provide new insights into DPN pathogenesis.

Other immune-related genes of interest were *GNAS* and *MAPK8IP3*. *GNAS*, a complex imprinted locus with multiple gene products, including the G protein α-subunit G_s_α [52], was hypermethylated and downregulated. *GNAS* is an interesting finding because it is critical for peripheral nerve myelination [53] and modulates lipid and glucose metabolism [54, 55], which are both dysregulated in experimental and clinical DPN [10, 14, 40]. We also detected hypomethylated and upregulated *MAPK8IP3,* a scaffold protein for c-Jun N-terminal kinase (JNK), in sural nerves of patients with higher HbA1c. In addition to its emerging role in nerve degeneration and axonal neuropathy [38], MAPK8IP3 may be an important player in the progression of human DPN through its close interaction with Toll-like receptor 4 (TLR4) and JNKs [56]. We recently demonstrated a role for TLR4 in immune-mediated inflammation in murine DPN [57]. Additionally, JNK is a key signaling pathway promoting inflammation and insulin resistance in diabetic complications, including DPN [40, 58]. Thus, our results support a role for epigenetic mechanisms in regulating immune-associated genes such as *PLCG2*, *GNAS*, and *MAPK8IP3* and suggest that they may be potential disease-modifying therapeutic targets in human DPN.

Another mechanism thought to contribute to DPN is ECM remodeling [59], and we have previously reported transcriptomic changes in the composition and function of the ECM pathways in DPN [9, 14]. In the current study, the overlapping genes between DMGs and DEGs were enriched with biological functions related to locomotion/cell migration and ECM-receptor interaction, consistent with the previous findings [9, 14]. In particular, we showed that the hypomethylation of tissue inhibitor of metalloproteinase 2 (*TIMP2*) was associated with reduced gene expression. TIMP2 regulates the activity of matrix metalloproteinase 2 (MMP2), and the MMP2/TIMP2 ratio is essential for maintaining ECM integrity [60]. An imbalance between MMP2 and TIMP2 contributes to ECM accumulation and fibrosis in diabetic nephropathy [61], an effect that may be mediated at least in part by TGF-β [62], which was also dysregulated in the present study. Additionally, the MMP2/TIMP2 axis modulates sciatic nerve ECM during nerve repair [63] and an impaired MMP2/TIMP2 axis has been implicated in the pathogenesis of DPN [64]. Given its prominent role in the regulation of nerve ECM, it is not surprising that our gene interaction network revealed that *TIMP2* was highly connected to members of the collagen family, such as collagen type VI, α3 (*COL6A3*), which has also been associated with fibrosis and inflammation in diabetic nephropathy [65]. While we speculate that alteration of *TIMP2* and *COL6A3* in human DPN results in ECM remodeling, further investigation will determine how these changes impact nerve function in diabetes.

Impaired insulin signaling in DPN may induce myelination deficits in Schwann cells and insulin resistance in sensory neurons [66, 67]. Our results show that insulin signaling pathway genes such as *IRS2* are differentially methylated and that *IGF-I* is downregulated, indicating impaired insulin signaling in the sural nerves of patients with higher HbA1c. Insulin and IGF-I both signal through the PI3K-Akt pathway, which in turn exerts multiple cellular actions through downstream effectors, including the mammalian target of rapamycin complex 1 (mTORC1) [68]. PI3K-Akt-mTORC1 signaling is heavily implicated in Schwann cell lipid synthesis, a critical mechanism for myelination [66], whose disruption is associated with impaired nerve function [69]. Consistent with these findings, our KEGG-based analysis revealed a dysregulated PI3K-Akt pathway at both the DNA methylation and gene expression levels, and suggests that DNA methylation may be a new mechanism for regulating PI3K-Akt in peripheral nerves. Although the mTORC1 pathway is not differentially expressed in this cohort, preliminary evidence from our group supports the involvement of mTORC1 in T2D-mediated nerve injury (data not shown), and future studies will elucidate the interaction between PI3K-Akt and mTORC1 in the context of DPN.

*Thy1* and *PTGS1* were the genes selected for RTq-PCR validation because of their relevance to known nerve injury mechanisms in DPN [37, 39]. *Thy1* and *PTGS1* were hypomethylated within the intergenic region and downregulated in gene expression, which is expected when methylation occurs outside the promoter region [22]. Chen et al. have shown that *Thy1* levels are reduced following nerve crush and return to near control levels after nerve repair [70]. While the type of nerve insult is different in our study (metabolic versus acute), our results also show that *Thy1* is downregulated following injury, supporting the idea that this gene is a negative regulator of neurite outgrowth. *PTGS1* encodes cyclooxygenase 1 (COX-1), a modulator of inflammation, which influences neuropathic pain in dorsal root ganglia and spinal neurons [39, 71]. Inhibiting COX-1 in streptozotocin-induced diabetic rats attenuates hyperalgesia [72]. The hypomethylation-dependent reduction in *PTGS1* gene expression in the sural nerves of patients with higher HbA1c suggests it is involved in DPN pathogenesis, supports our findings of a role for inflammation in DPN, and warrants further investigation.

## Conclusions

We profiled the sural nerve methylome and transcriptome in human DPN to identify changes in molecular mechanisms underlying disease pathogenesis. We show that HbA1c levels are associated with transcriptomic changes and pathways which modulate important molecular functions, such as the immune response and axon guidance. Our functional and network analyses of integrated epigenetic and transcriptomic data revealed regulation of some of these pathways by DNA methylation, a potentially reversible mechanism linking genetics and lifestyle factors. We found that immune response, ECM-receptor interaction, and PI3K-Akt signaling pathways are under epigenetic control and may play a crucial role in DPN development. Although the exact mechanisms are yet to be elucidated, our results suggest that optimal glycemic control is one of the important factors in maintaining epigenetic homeostasis and nerve function.

## Methods

### Sample collection and preparation

Human sural nerve biopsies were collected during a previous 52-week double-blind placebo-controlled clinical trial of acetyl-L-carnitine for treating DPN [28, 29]. The trial design and selection criteria were described previously [29]. Briefly, the trial included adult male and female subjects diagnosed with T1D or T2D for at least 1 year and with an HbA1c > 5.9%, with clinically evident DPN, defined by at least 2 neurological findings, including clinical symptoms or abnormal electrophysiological tests (nerve conduction velocity or vibration perception threshold) [73, 74]. Patients with non-diabetic causes of neuropathy, complicating diseases (such as HIV or significant cardiac or hepatic disorders), and alcohol or drug abuse were excluded.

A sural nerve biopsy was collected from the distal calf at the time patients enrolled and another biopsy was collected from the opposite leg after 52 weeks of treatment. Sural nerve myelinated fiber density (MFD) is a reliable morphological criterion for DPN diagnosis [75]. Based on percent myelinated fiber density change (%delta-MFD) over 1 year, these patients were divided into three groups (denoted as regenerator, intermediator, and degenerator) in the previous analysis [30]. For the current study, a total of 78 patients with T2D were selected for methylome and transcriptome profiling; patient characteristics are given in Additional file 2: Table S1. Different from previous analyse s[30], these 78 patients were segregated into groups 1 and 2 using an unbiased clustering analysis (see below methods). The University of Michigan Institutional Review Board for Human Subject Research approved the use of human sural nerve samples.

### Genome-wide gene expression and methylation profiling

DNA and RNA were extracted from human sural nerves using the Qiagen AllPrep DNA/RNA Mini Kit (Qiagen, Valencia, CA, USA) according to the manufacturer’s protocol. DNA and RNA quantity was assessed using a Qubit fluorometer, and the quality was evaluated using a 2100 Bioanalyzer (Agilent Technologies, Santa Clara, CA).

Samples with RNA integrity numbers ≥ 8 were prepared for RNA-sequencing (RNA-seq) using the Illumina TruSeq mRNA Sample Prep v2 kit (Illumina, San Diego, CA, USA). Approximately 90 million 50-base pair (bp) paired-end reads per sample were obtained on an Illumina HiSeq 2500 system (Illumina, San Diego, CA). RNA-seq was performed by the University of Michigan DNA Sequencing Core (http://seqcore.brcf.ed.umich.edu).

RRBS was performed by the University of Michigan Epigenomics Core as previously described [76]. Briefly, DNA was digested with the *MspI* restriction enzyme and purified using phenol:chloroform extraction and ethanol precipitation, blunt-end digested, phosphorylated, and ligated into an adapter duplex with methylated cytosines. The ligated fragments were cleaned and size selected by an agarose gel. Bisulfite conversion was performed for selected fragments followed by PCR amplification and cleanup using AMPure XP beads. The Qubit assay and Agilent’s High Sensitivity D1000 assay were used to quantify the libraries, which were then sequenced on the Illumina HiSeq 2500 platform to obtain approximately 90 million 50 bp single-end reads per sample.

### Sequencing data analysis

#### Quality filtering and read mapping

For the RNA-seq data, low-quality (Q < 30) reads and sequencing adapters were removed with Trimmomatic [77] and the quality of raw reads was assessed with FastQC (version 0.11.5, Babraham Bioinformatics, UK). Clean reads were then mapped to the human reference genome hg19 Refseq using Hisat2 [78]. FeatureCounts [79] was used to count reads mapped to individual genes and only uniquely mapped reads were used in the counting step. Genes with zero expression across all samples were omitted from the correlation and differential expression analysis. Fragments per kilobase of exon per million mapped reads (FPKM) was calculated to represent gene expression levels.

Similarly for the RRBS data, quality control of the RRBS data was performed using FastQC (version 0.11.5, Babraham Bioinformatics, UK), and low-quality reads were removed with Trim Galore (version 0.5.0, Babraham Bioinformatics, UK). Then, trimmed reads were aligned and mapped using Bismark [80] to the human hg19 reference genome. The percentage methylation level was calculated by #C/(#C + #T), where #C is the number of methylated reads and #T is the unmethylated reads. Then, only the CpG sites with a read coverage > 10, a quality score > 20, and appeared at least in 10 samples among each group, using the parameter “min.per.group = 10,” were kept for downstream analysis.

#### Unbiased sample clustering based on RNA-seq data

The RNA-seq data was normalized by DESeq2 R package using the default parameters [81]. Unsupervised hierarchical clustering and principal component analysis on the normalized expression values were used to examine the overall similarity among the samples, which identified three sample groups with high similarity. We also determined whether clinical factors (Additional file 2: Table S1) were significantly associated with the three groups using multifactorial logistic regression analyses.

#### Differential expression and methylation analyses

Differentially expressed genes (DEGs) were identified using DESeq2 [81] and genes with a Benjamini-Hochberg adjusted *p* value < 0.01 were deemed as DEGs. Differentially methylated CpGs (DMCs) were defined as a CpG site with a methylation difference of > 15% and a false discovery rate adjusted *p* value (*q* value) < 0.01 between group 1 and group 2. DMCs were then annotated based on genes and CpG island (CGi) features. Each DMC was mapped to a gene, having the shortest distance from its transcript starting site to the DMC. These mapped genes were defined as differentially methylated genes (DMGs).

#### Functional enrichment analysis

To identify and compare the overrepresented biological functions, enrichment analysis was performed using a hypergeometric test with our in-house R analysis package richR (http://github.com/hurlab/richR). Kyoto Encyclopedia of Genes and Genomes (KEGG) pathways, Gene Ontology (GO) terms, Reactome pathway (https://reactome.org), and Disease Ontology (DO: http://disease-ontology.org) terms were used in the enrichment analysis, and the terms with Benjamini-Hochberg corrected *p* values < 0.05 were deemed as significantly overrepresented biological functions in each DEG set. To reduce redundancy in the GO enrichment results, additional term clustering was performed using the clustering parameter kappa > 0.5, which included GO clusters with a minimum of 5 GO terms, as implemented in the Database for Annotation, Visualization and Integrated Discovery (DAVID) [33, 82]. The same functional enrichment analysis was performed for the DMG sets with a nominal *p* value < 0.05 as the cutoff value. Dot plots with the top 20 most significant terms were generated.

### RT-qPCR validation

Two shared genes between DMGs and DEGs (*Thy1* and *PTGS1*) were selected for validation by quantitative real-time polymerase chain reaction (RT-qPCR). RNA was isolated from sural nerve biopsies using RNeasy fibrous tissue mini kit (Qiagen, Valencia, CA, USA) from group 1 (*n*  =  9–10) and group 2 (*n*  =  10), which was used as the relative control group (set to 100%). Reverse transcription was performed using iScript Supermix (Bio-Rad Laboratories, Hercules, California). qPCR reactions were carried out using sequence-specific TaqMan™ probes (ThermoFisher/Applied Biosystems) for *Thy1* (Hs06633377_s1) and *PTGS1* (Hs00377726_m1) in an Applied Biosystems StepOneTM RT-PCR system. Using the 2^−ΔΔCT^ method, tyrosine 3-monooxygenase/tryptophan 5-monooxygenase activation protein (YWHAZ) was used as the endogenous control and group 2 as the relative control. Statistically significant differences were calculated using a Student’s *t* test with the GraphPad Prism 7 software (GraphPad Software Inc.).

## Supplementary information

**Additional file 1: Figure S1.** Sample distribution based on transcriptomic data. **Figure S2.** MA plot showing the top 50 DEGs in Group 1 versus Group 2. **Figure S3.** Gene expression patterns of the most significant 100 DEGs between Group 1 and Group 2. **Figure S4.** Functional enrichment analysis of DEGs by Reactome and DO. **Figure S5.** Functional enrichment analysis of DMGs by Reactome and DO. **Figure S6.** Functional enrichment analysis of overlapping DEGs and DMGs in the same direction.

**Additional file 2: Table S1.** Patient characteristics. **Table S2.** RNA-seq & RRBS read quality and mapping summary. **Table S3.** Differentially expressed genes and their annotation. **Table S4.** KEGG enrichment analysis of DEGs. **Table S5.** DEG GO Clustering. **Table S6.** Reactome enrichment analysis of DEGs. **Table S7.** Disease ontology enrichment analysis of DEGs. **Table S8.** Differentially methylated CpG sites and their annotation. **Table S9.** KEGG enrichment analysis of DMGs. **Table S10.** DMG GO Clustering. **Table S11.** Reactome enrichment analysis of DMGs. **Table S12.** Disease ontology enrichment analysis of DMGs. **Table S13.** DEG & DMG overlap genes information.

## Data Availability

The datasets generated and/or analyzed during the current study are available in the NCBI Gene Expression Omnibus (Accession ID: GSE148061; token for reviewer’s access: “cfytwymepnwjdqf” without quotes).

## Abbreviations

DAVID: Database for Annotation, Visualization and Integrated Discovery
DEG: Differentially expressed gene
DMC: Differentially methylated CpG site
DMG: Differentially methylated gene
DO: Disease Ontology
DPN: Diabetic peripheral neuropathy
GO: Gene Ontology
KEGG: Kyoto Encyclopedia of Genes and Genomes
MFD: Myelinated fiber density
RNA-seq: RNA-sequencing
RRBS: Reduced representation bisulfite sequencing
STRING: Search Tool for the Retrieval of Interacting Genes
TSS: Transcription starting site
