# Correction to: Genome-wide profiling of DNA methylation and gene expression identifies candidate genes for human diabetic neuropathy

**DOI:** 10.1186/s13148-020-00922-5

**Published:** 2020-08-27

**Authors:** Kai Guo, Stephanie A. Eid, Sarah E. Elzinga, Crystal Pacut, Eva L. Feldman, Junguk Hur

**Affiliations:** 1 Department of Biomedical Sciences, School of Medicine and Health Sciences, University of North Dakota, 1301 North Columbia Rd. Stop 9037, Grand Forks, ND 58202-9037 USA; 2 Department of Neurology, School of Medicine, University of Michigan, Ann Arbor, MI 48109 USA

**Correction to: Clin Epigenet 12, 123 (2020)**

**https://doi.org/10.1186/s13148-020-00913-6**

Following publication of the original article [1], we were notified of a mistake in the way the author corrections have been implemented: a few random numbers had been incorrectly assigned to the second column in Fig. 5A. These have now been removed.

The original article has been corrected.
