# Supplementary material for: Genome-wide profiling of DNA methylation and gene expression identifies candidate genes for human diabetic neuropathy
Source: Clin Epigenetics. 2020 Aug 12;12:123. doi: 10.1186/s13148-020-00913-6 (PMC7425575; doi:10.1186/s13148-020-00913-6)
Supplement: Supplementary file 1 — Additional file 1: Figure S1. Sample distribution based on transcriptomic data. Figure S2. MA plot showing the top 50 DEGs in Group 1 versus Group 2. Figure S3. Gene expression patterns of the most significant 100 DEGs between Group 1 and Group 2. Figure S4. Functional enrichment analysis of DEGs by Reactome and DO. Figure S5. Functional enrichment analysis of DMGs by Reactome and DO. Figure S6. Functional enrichment analysis of overlapping DEGs and DMGs in the same direction. [file 13148_2020_913_MOESM1_ESM.docx]

**Additional file 1: Figure S1.** **Sample distribution based on transcriptomic data.** (A) Hierarchical clustering was performed using the RNA-seq expression data. The dendrogram is colored based on similarity in the transcriptomic data among the samples, while the leaf (sample ID) represents the biological groups. In the initial analysis, these biological groups were classified based on changes in MFD after 52 weeks and consisted of three groups: 1) regenerators showing an increase, 2) degenerators showing a decrease, and 3) intermediators showing no change (B) Principal component analysis was applied to the three data-driven groups, identified in the color-coded clustering analysis. Each dot represents an individual sample with the sample ID shown. Node color corresponds to the group identified by hierarchical clustering.

**(A)**


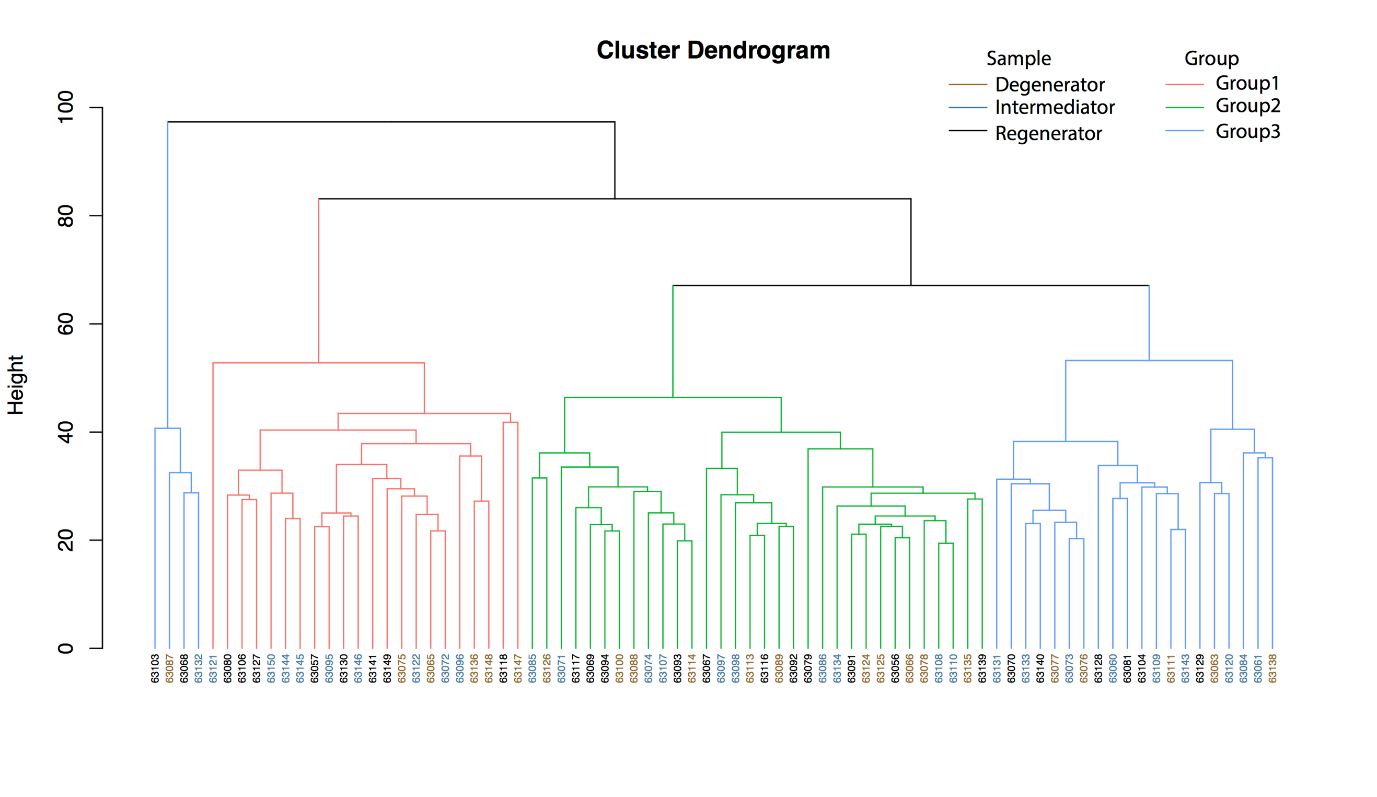


**(B)**


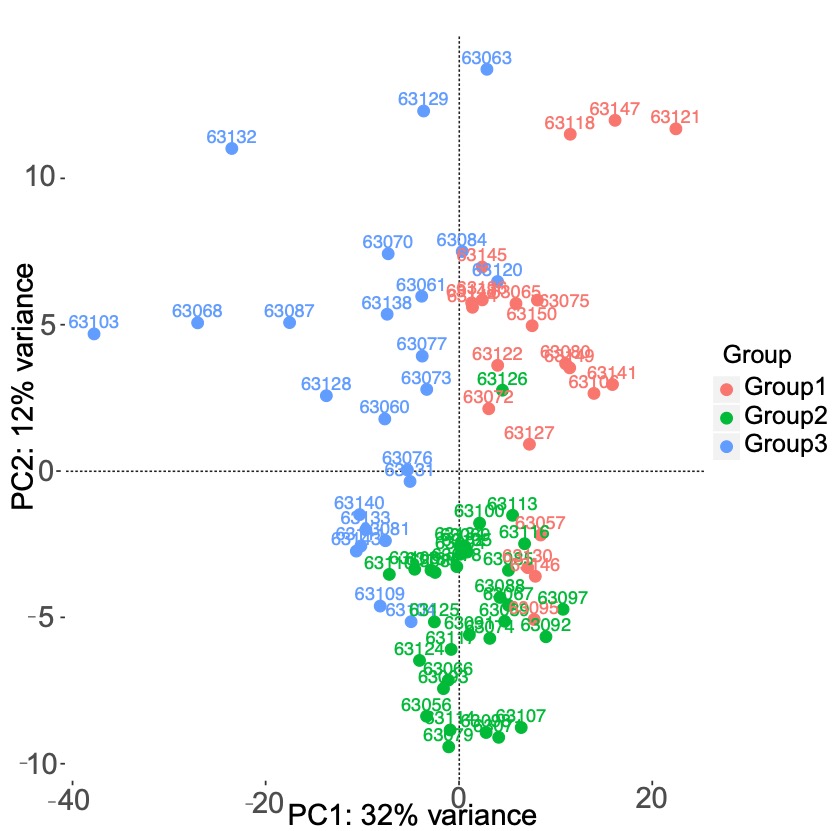


**Additional file 1:** **Figure S2. MA plot showing the top 50 DEGs in Group 1 versus Group 2.** Each dot corresponds to an individual gene and the 50 genes with the most significant changes are labeled. Dots colored in red and blue correspond to the DEGs with a minimum 2-fold up- or downregulation, respectively.

**Additional file 1:** **Figure S3. Gene expression patterns of the most significant 100 DEGs between Group 1 and Group 2.** Genes (rows) were clustered based on their expression profiles.

**Additional file 1:** **Figure S4. Functional enrichment analysis of DEGs by Reactome and DO.** The 20 most significantly enriched biological functions using Reactome (A) and DO (B) are illustrated in dot plots. Rich Factor refers to the proportion of DEGs belonging to a specific term. Node size (Gene number) refers to the number of DEGs within each term and node color indicates the level of significance (-log_10_p-value).

**Additional file 1:** **Figure S5. Functional enrichment analysis of DMGs by Reactome and DO.** The 20 most significantly enriched biological functions using Reactome (A) and DO (B) are illustrated in dot plots. Rich Factor refers to the proportion of DMGs belonging to a specific term. Node size (gene number) refers to the number of DEGs within each term and node color indicates the level of significance (-log_10_p-value).

**Additional file 1: Figure S6. Functional enrichment analysis of overlapping DEGs and DMGs in the same direction.** KEGG enrichment analysis was done for the overlapping genes (Hypo-Down and Hyper-Up groups). Dot plot includes the significantly enriched KEGG pathways.

**
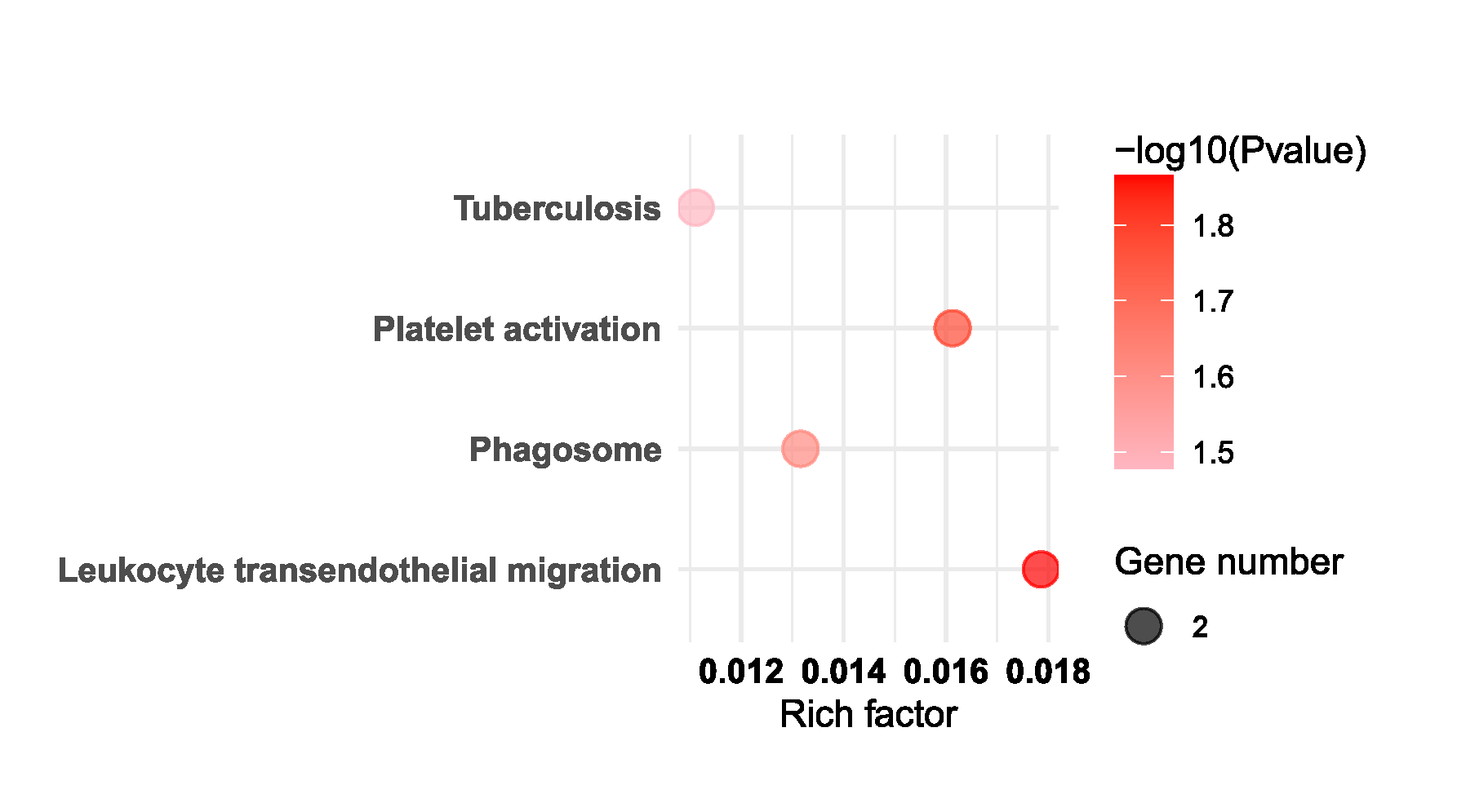
**
